# Predictors of web-based follow-up response in the Prevention of Low Back Pain in the Military Trial (POLM)

**DOI:** 10.1186/1471-2474-12-132

**Published:** 2011-06-13

**Authors:** John D Childs, Deydre S Teyhen, Joshua J Van Wyngaarden, Brett F Dougherty, Bryan J Ladislas, Gary L Helton, Michael E Robinson, Samuel S Wu, Steven Z George

**Affiliations:** 1 US Army-Baylor University Doctoral Program in Physical Therapy (MCCS-HMT), Army Medical Department Center and School, 3151 Scott Rd., Rm. 2307, Fort Sam Houston, TX 78234, USA; 2 Department of Clinical and Health Psychology, PO Box 100165, Health Sciences Center, University of Florida, Gainesville, FL 32610, USA; 3 Department of Biostatistics, 1329 SW 16th St., Rm. 5231, PO Box 100177, University of Florida Gainesville, FL 32610-0177, USA; 4 Department of Physical Therapy, Center for Pain Research and Behavioral Treatment, PO Box 100154, University of Florida, Gainesville, FL 32610-0154, USA

## Abstract

**Background:**

Achieving adequate follow-up in clinical trials is essential to establish the validity of the findings. Achieving adequate response rates reduces bias and increases probability that the findings can be generalized to the population of interest. Therefore, the purpose of this study was to determine the influence of attention, demographic, psychological, and health status factors on web-based response rates in the ongoing Prevention of Low Back Pain in the Military (POLM) trial.

**Methods:**

Twenty companies of Soldiers (n = 4,325) were cluster randomized to complete a traditional exercise program including sit-ups (TEP) with or without a psychosocial educational program (PSEP) or a core stabilization exercise program (CSEP) with or without PSEP. A subgroup of Soldiers (n = 371) was randomized to receive an additional physical and ultrasound imaging (USI) examination of key trunk musculature. As part of the surveillance program, all Soldiers were encouraged to complete monthly surveys via email during the first year. Descriptive statistics of the predictor variables were obtained and compared between responders and non-responders using two sample t-tests or chi-square test, as appropriate. Generalized linear mixed models were subsequently fitted for the dichotomous outcomes to estimate the effects of the predictor variables. The significance level was set at .05 a priori.

**Results:**

The overall response rate was 18.9% (811 subjects) for the first year. Responders were more likely to be older, Caucasian, have higher levels of education and income, reservist military status, non smoker, lower BMI, and have received individualized attention via the physical/USI examination (p < .05). Age, race/ethnicity, education, military status, smoking history, BMI, and whether a Soldier received the physical/USI examination remained statistically significant (p < .05) when considered in a full multivariate model.

**Conclusion:**

The overall web based response rate during the first year of the POLM trial was consistent with studies that used similar methodology, but lower when compared to rates expected for standard clinical trials. One year response rate was significantly associated with demographic characteristics, health status, and individualized attention via additional testing. These data may assist for planning of future trials that use web based response systems.

**Trial Registration:**

This study has been registered at reports at http://clinicaltrials.gov (NCT00373009).

## Background

Achieving adequate follow-up in clinical trials is essential to establish the validity of study findings and reduce bias, helping to insure that the findings can be generalized to the population of interest and more accurately inform clinical decision-making. Studies with low follow-up rates potentially confound interpretation of the results since subjects who drop out may be materially different from those who complete the study (i.e. attrition bias)[1]. Low subject response rates can further threaten external validity by impairing the ability of researchers to make clear scientific conclusions based on their data [1]. According to Straus et al, follow-up rates that exceed 95% minimize the potential for attrition bias to exist whereas follow-up rates lower than 80% pose a threat to external validity [2,3]. Even small losses to follow-up can bias a study's results if few individuals have the outcome of interest. Collectively, these issues make it imperative that clinical trials be conducted in a manner to maximize retention.

One of the most commonly reported factors to be associated with improving retention and follow-up is the attention afforded to subjects during their participation in the study [4]. Dias et al found that increased attention in the form of staff friendliness, responsiveness, and subject encouragement positively influenced long-term follow-up, with retention rates of 98.5% for their 3 year study [4]. Alternatively, Loftin et al found that failing to follow-up with subjects consistently and develop caring and trusting relationships with study participants negatively impacted retention [5]. One might presume that increased attention at an individual level (ie, physical examination, interview, etc.) might translate into improved retention and follow-up compared to group-based attention (ie, educational class) because of the potential to form a deeper connection with subjects in a one-to-one environment compared to a group setting. The experimental groups in the Loftin studies received both group and individual attention through dietary classes and weekly phone calls, respectively, hence they were unable to determine whether increased individual attention is superior to group-based attention [5]. Further studies are needed to determine the influence of attention, especially analyses that allow for comparison of different forms of attention.

A number of other factors have also been purported to positively influence long-term follow-up. These include age over 60, those with lower baseline self-efficacy, and a participant's belief in the merits of the study [6]. Loftin et al found that subjects with higher rates of follow-up had stronger beliefs about the extent to which the study significantly contributed to the community and the advancement of science [5]. Conversely, a number of factors have been shown to negatively influence retention in trials. Janson et al conducted a study on 35 subjects who had voluntarily withdrawn from a large, multi-center randomized trial [7]. The primary factor found to be associated with decreased retention was a perceived lack of sensitivity on the part of the research staff. There were also a few demographic characteristics commonly associated with subject withdrawal to include younger individuals and ethnic minorities. While these factors tended to influence drop-out rates, they did not achieve statistical significance secondary to lack of power as a result of the small sample size of 35 [7]. Other studies have reinforced the notion that demographic factors are not highly predictive of drop-out rates. For example, a large RCT with over 2,311 subjects failed to detect a relationship between BMI, sex, ethnicity, and retention at one year follow up [6].

Further research is needed to identify potentially important factors that influence follow-up rates. Then these factors could be appropriately considered when designing clinical trials. As part of the ongoing Prevention of Low Back Pain in the Military (POLM) trial, we utilized a novel web-based surveillance system to track subject response rate and record incidence and severity of low back pain (LBP) episodes among a group of geographically dispersed Soldiers in the U.S. Army over a 2-year period [8]. As part of the trial, we had access to many baseline variables previously found to be associated with follow-up rates in trials. Therefore, the purpose of this secondary analysis was to determine predictors during the first year of web-based response rates in the POLM trial. We hypothesized that subjects receiving increased attention via a randomly selected education program or physical examination session would have higher follow-up rates than those receiving less attention. We also sought to determine the influence of various demographic, psychological, and health status factors on web-based response rates.

## Methods

### Design Overview

This study reports a planned secondary analysis in the Prevention of Low Back Pain in the Military clinical trial (NCT00373009) which has been registered at http://clinicaltrials.gov[8]. Consecutive subjects entering a 16-week training program at Fort Sam Houston, TX to become a combat medic in the U.S. Army were considered for participation. In the primary trial, 20 companies of Soldiers were cluster randomized to complete one of 4 training programs: a traditional exercise program including sit-ups (TEP) with (n = 945) or without (n = 1,212) a psychosocial educational program (PSEP) or a core stabilization exercise program (CSEP) with (n = 1,049) or without PSEP (n = 1,089)[9,10]. Subjects in each of the 4 groups performed the assigned exercise program in a group setting under the direct supervision of their drill instructors as part of daily unit physical training [8,11,12]. Subjects are currently being followed monthly for two years using a web-based surveillance system to record incidence and severity of subsequent LBP episodes. However, the primary trial results are not yet available. For this analysis, we collapsed the study into a single cohort for the purpose of determining predictors of 1-year response rates to the web-based follow-up survey.

### Setting and Participants

**Figure 1:**
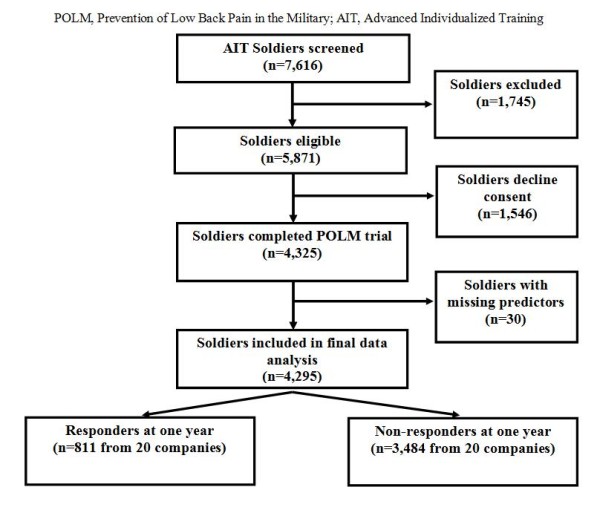
**Flow diagram for subject recruitment and email responders one year after the conclusion of the study**.

Research staff at Fort Sam Houston, Texas introduced the study to individual companies of Soldiers and obtained written informed consent. Refer to Figure 1 for a flow diagram describing the number of companies and Soldiers considered for this trial, eventually enrolled into the trial, and completed the 1-year web-based follow-up survey, as per the Consolidated Standards of Reporting Trials (CONSORT) guidelines [9]. All subjects were recruited during a training orientation session attended by all Soldiers as part of their in-processing for medic training. For 8 consecutive months subjects were screened for eligibility according to the inclusion/exclusion criteria. Subjects were required to be 18-35 years of age (or 17 year old emancipated minor), participating in training to become a combat medic, and be able to speak and read English. Subjects with a prior history of LBP were excluded. A prior history of LBP was operationally defined as LBP that limited work or physical activity, lasted longer than 48 hours, and caused the subject to seek health care. Subjects were also excluded if they were currently seeking medical care for LBP; unable to participate in unit exercise due to injury in foot, ankle, knee, hip, neck, shoulder, elbow, wrist, or hand; had a history of fracture (stress or traumatic) in proximal femur, hip, or pelvis; were pregnant; or if they had transferred from another training group. Other possible exclusions included Soldiers who were being accelerated into a Company already randomized and recruited for participation in the Prevention of Low Back Pain in the Military trial or Soldiers who were being re-assigned to an occupational specialty other than a combat medic.

### Ethics Approval

The institutional review boards at the Brooke Army Medical Center (San Antonio, TX) and the University of Florida (Gainesville, FL) granted approval for this project. All subjects provided written informed consent prior to their participation.

### Potential Predictors of Response Rates to the Web-based Survey

Select demographic characteristics, psychological variables, health status and physical activity, injury status, and attention/relationship effect variables were considered as potential predictors of 1-year response rates on the web-based follow-up survey. These measures were collected at baseline using a variety of commonly utilized and previously validated self-report questionnaires and physical examination procedures performed by research personnel unaware of randomization assignment at baseline. All measures were scored in a masked manner by computer algorithm.

### Demographic Characteristics

Demographic characteristics were considered as both a) potential predictors of response rate and b) risk adjustment variables. These characteristics included age, sex, race/ethnicity, level of education, income, length of service, military status, and assigned Company drill instructors.

### Psychological Variables

The Back Beliefs Questionnaire (BBQ) is a previously validated self-report questionnaire used to quantify beliefs about the likely consequences of having LBP. Higher BBQ scores are indicative of better LBP beliefs and indicate the potential of a better ability to cope with LBP [13]. The State-Trait Anxiety Questionnaire (STAI) and Beck Depression Inventory (BDI) were used to measure negative affect from generalized anxiety and generalized depression, respectively [13]. Higher scores on these indices were indicated of higher anxiety and depressive symptoms. Nine items from the Fear of Pain Questionnaire (FPQ-III) were used to measure fear about specific situations that normally produce pain [13]. Higher scores on the fear indices indicated higher general fear of pain and fear of low back pain.

### Health Status and Physical Activity

The Medical Outcomes Survey 12-Item Short-Form Health Survey (SF-12) was used as a self-report of health status for physical and mental function. The physical and mental component summary scales (PCS and MCS) were reported individually in this study because they are valid estimates of physical and mental health [13].

As part of the intake questionnaire, Soldiers were queried as to their level of physical activity prior to entering training. Specifically, Soldiers were asked to report how many days per week on average they performed at least 30 minutes of exercise and how many years over the course of their lifetime they have consistently exercised at least 3 days per week prior to entering training. Soldiers were queried regarding their smoking status, and their body mass index (BMI) was calculated [8].

#### Attention/Relationship Effect

##### Psychosocial Educational Program

Soldiers who were randomized to PSEP (n = 1994) completed an educational session within a group setting during the first 14 days of entering training. The session consisted of an interactive seminar designed by the POLM investigative team and was implemented by study personnel. The overall goal of the 45 minute session was to emphasize current scientific evidence on LBP based on biopsychosocial principles that promote healthy beliefs about LBP. The seminar covered topics related to the favorable natural history of LBP, lack of definitive anatomical causes of LBP, the importance of returning to normal activity, and decreasing fear-avoidance beliefs and pain catastrophizing when experiencing LBP. Soldiers were informed why educational information on best LBP coping strategies was important despite the fact they did not currently have LBP. After the seminar, Soldiers participated in a question and answer session and were issued The Back Book [8]. The Back Book was used as the educational supplement because of our prior experience with it in a physical therapy clinical trial and its prior association with positive shifts in patient LBP beliefs [13].

##### Physical Examination of Trunk Musculature

Because it would be time and cost prohibitive to perform an extensive physical examination on all subjects in a trial this large, a subgroup of Soldiers from each company (n = 371) were randomized to receive additional testing in the form of a physical and ultrasound imaging (USI) examination of key trunk musculature. The physical examination consisted of measuring low back range of motion, straight leg raise, and bilateral hip range of motion measurements. Soldiers also completed 4 trunk muscle endurance tests (extension, flexion, and bilateral side supports) by determining how long a specific position could be maintained. Separately, a USI examination was performed which included assessment of the lateral abdominal muscles (transverse abdominus, internal and external oblique muscles) during an active straight leg raise and symmetry of the multifidi muscles [8]. The examination required approximately 2 hours. Soldiers who received the physical/USI examination and/or receive the PSEP were classified as having received additional attention for the purpose of assessing the potential for increased attention to influence response rates.

### Web-based Follow-up Surveys

At the end of the initial 12 weeks of training, Soldiers were trained in a computer lab on how to use the web-based surveillance system to complete the monthly follow-up surveys. The purpose of the follow-up surveys was to record incidence and severity of subsequent LBP episodes in the previous calendar month. Access to the web-based surveillance system was prompted by an email, which was sent to the Soldier's official military email address on the 1^st ^of each month. The web-based survey started with an email prompting to visit the study hosted, confidential, secure web-site. Once the website was accessed, Soldiers were asked one initial screening question - "have you had any back pain in the past 30 days?" A "no" answer ended the survey and Soldiers were thanked for their participation. A "yes" answer prompted the Soldiers to complete an additional set of 46 items about the back pain episode including duration, impact on work activities, whether health care was sought, and response to standard questionnaires (ie, NPRS, ODQ, FABQ, and PCS). Soldiers were provided their login credentials (user name and password) during the initial training session at the end of the 12-week trial. Login credentials were also provided in the monthly email reminders. If a Soldier did not respond to the first email, an additional email was sent on the 3^rd ^of the month, and again on the 7^th ^of the month if the Soldier still had not responded.

## Data Analysis

The primary dependent variable for this paper was the dichotomous outcome of whether a Soldier responded to any one of the 12 monthly surveys. The independent variables considered as potential predictors of response rate included psychological variables (BDI, FPQ, BBQ, STAI), health status and physical activity (SF-12 PCS and MCS total, smoking status, level of physical activity, BMI), and the attention/relationship effect (received physical/USI examination or PSEP). Potential effects of additional attention in the form of an individualized examination and group attention from the PSEP were examined separately. Other explanatory variables of interest and for risk adjustment included demographic characteristics such as age, sex, race/ethnicity (White/Caucasian, Black/African American, Hispanic, and others), level of education (College or more, Some college, High school or less), income ($>20,000 or more, length of service (<5 months, 5 months-1 year, >1 year), military status (Active duty, Reservist, or National Guard), and assigned Company drill instructors.

Descriptive statistics of the demographic and clinical variables were compared between the responders and non-responders using two sample t-tests or chi-square test, as appropriate. A generalized linear mixed model was then fitted for the dichotomous outcome to estimate the effects of potential predictors and the other explanatory variables listed above. A random company effect was included in the models to accommodate for the correlation among Soldiers within the same company. Furthermore, to assess the response difference over time, we fitted a second generalized linear mixed model using the longitudinal binary outcomes of whether a Soldier responded to each one of the 12 monthly surveys as dependent variable, with quadratic time effect in addition to the same predictor/explanatory variables as in the first model. The significance level was set at .05 a priori, and all analyses were performed with the use of SAS software, version 9.1.

## Results

Among the 4,325 Soldiers who completed POLM trial, 4,295 Soldiers (99.3%) had complete data in all predictor variables and included in the final analyses (Figure 1). Among the 4,295 Soldiers, 71% were male, 72% were White/Caucasian, 55% had at least some college or more education, 51% had $20,000 or more household income, 63% had been enlisted in the Army for less than 5 months, and 15% for more than 1 year. The study population had a mean age of 22.0 years (SD = 4.2) (Table 1). The overall response rate to the web-based survey was 18.9% (811 subjects) for the first year of the POLM trial.

**Table 1:** Statistical analysis of web-based responders and non-responders via during first year follow up from POLM study

Variables	Overall (n = 4,295)	No Response (n = 3,484)	Response (n = 811)	P-value
Age	22.0 (4.2)	21.8 (4.1)	22.8 (4.7)	<.001

Gender				
Female	1233 (28.7%)	978 (28.1%)	255 (31.4%)	.056
Male	3062 (71.3%)	2506 (71.9%)	556 (68.6%)	

Race/Ethnicity				
African American	444 (10.3%)	367 (82.7%)	77 (17.3%)	
				.043
Caucasian	3,094 (72.0%)	2,508 (81.1%)	586 (18.9%)	
Other	757 (17.7%)	609 (80.4%)	148 (19.6%)	

Education				
High school or lower	1,952 (45.4%)	1,667 (85.4%)	285 (14.6%)	
Some college	1,955 (45.5%)	1,549 (79.2%)	406 (20.8%)	<.001
Graduated from college or higher	388 (9.0%)	268 (69.1%)	120 (30.9%)	

Income				
<$20,000	2,119 (49.3%)	1,750 (82.6%)	369 (17.4%)	
				.015
$20,000 or more	2,176 (50.7%)	1,734 (79.7%)	442 (20.3%)	

Military Status				
Active	2,518 (58.6%)	2,125 (84.4%)	393 (15.6%)	
				<.001
Reserve	1,777 (41.4%)	1,359 (76.5%)	418 (23.5%)	

Length of Service <5 months	2,684 (62.5%)	2,232 (83.2%)	452 (16.8%)	
5 months - 1 year	964 (22.4%)	750 (77.8%)	214 (22.2%)	<.001
> 1 year	647 (15.1%)	502 (77.6%)	145 (22.4%)	

Depression (BDI)	6.4 (6.6)	6.5 (6.7)	6.0 (6.1)	.039

Fear of Pain (FPQ)	18.1 (5.9)	18.0 (5.9)	18.2 (5.6)	.452

Back Beliefs (BBQ)	43.4 (7.1)	43.3 (7.0)	44.0 (7.4)	.005

Anxiety (STAI)	36.0 (9.2)	36.2 (9.2)	35.2 (9.1)	.004

Physical Health Status (PCS Total)	53.4 (5.1)	53.4 (5.1)	53.5 (5.2)	.400

Mental Health Status (MCS Total)	49.2 (8.6)	49.1 (8.7)	49.6 (8.0)	.099

Smoke Prior to Army				
No	2,756 (64.2%)	2,151 (78.0%)	605 (22.0%)	<.001
Yes	1,539 (35.8%)	1,333 (86.6%)	206 (13.4%)	

Company Instructor				
Alpha	621 (14.5%)	494 (14.2%)	127 (15.7%)	
Bravo	929 (21.6%)	766 (22.0%)	163 (20.1%)	
Charlie	607 (14.1%)	497 (14.3%)	110 (13.6%)	
Delta	957 (22.3%)	760 (21.8%)	197 (24.3%)	.256
Echo	660 (15.4%)	531 (15.2%)	129 (15.9%)	
Foxtrot	521 (12.1%)	436 (12.5%)	85 (10.5%)	

BMI	24.8 (3.2)	24.8 (3.2)	24.6 (3.2)	.027

Physical Examination				
No	3,924 (91.4%)	3,202 (81.6%)	722 (18.4%)	.009
Yes	371 (8.6%)	282 (76.0%)	89 (24.0%)	

Psychosocial Educational Program (PSEP)				
No	2,301 (53.6%)	1,871 (81.3%)	430 (18.7%)	
				.726
Yes	1,994 (46.4%)	1,613 (80.9%)	381 (19.1%)	

Exercise Group				
TEP only	1,212 (28.2%)	990 (28.4%)	222 (27.4%)	
TEP+PSEP	945 (22.0%)	767 (22.0%)	178 (21.9%)	
				.932
CSEP only	1,089 (25.4%)	881 (25.3%)	208 (25.6%)	
CSEP+PSEP	1,049 (24.4%)	846 (24.3%)	203 (25.0%)	

POLM, Prevention of Low Back Pain in the Military; BDI, Beck Depression Inventory; BBQ, Back Beliefs Questionnaire; STAI, State-Trait Anxiety Index; SF12, Medical Outcomes Survey 12-Item Short-Form Health Survey; BMI, Body Mass Index; PSEP, Psychosocial Educational Program. The p-values are based on t-tests or chi-square test, as appropriate.

**Figure 2:**
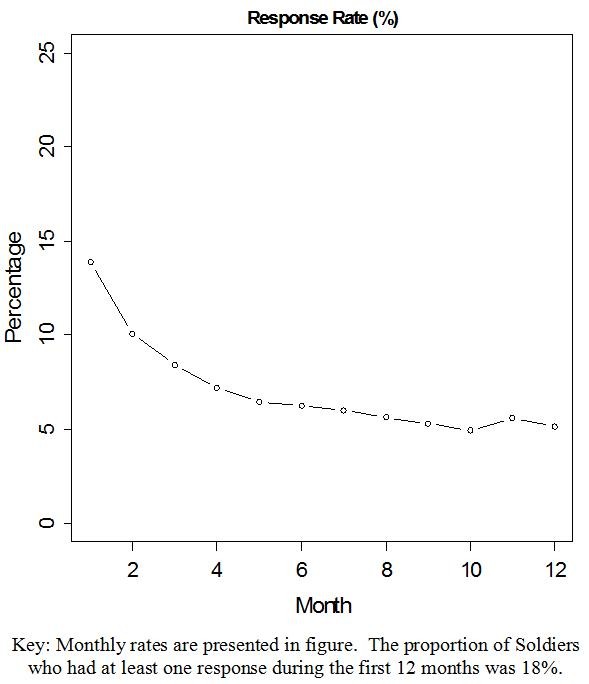
**Monthly response rate during the first year follow up**.

Non-responders and responders significantly differed in age, race/ethnicity, education, income, military status, length of service, depression, back beliefs, anxiety, health status, smoking history, BMI, and whether a Soldier received individual attention from the physical/USI examination (all with p < .05, Table 1). Based on the adjusted model (Table 2), the odds of response increased by 5% for every one year increase in age. Black/African American Soldiers had .76 times odds of response compared to White/Caucasian. Compared with Soldiers with college or higher education, the odds of response were .54 and .70 times for those with high school or less and those with some college education, respectively. Full-time active duty service members had .68 times odds of response compared to those from a Reserve or National Guard unit. The odds of response decreased by 3% for every one unit increase in BMI. Those who did not smoke had 1.69 times odds of response compared to those who smoked prior to entering the Army. In addition, those who did not receive the physical/USI examination had .70 times odds of response compared to those who received the examination. There was no difference in response rate based on whether Soldiers received group attention via the PSEP. The following factors: income, length of service, BDI, BBQ and SF-12 became statistically non-significant after adjusting the previously stated factors (Table 2). In addition, the above effects remained statistically significant in the second generalized linear mixed model that included the quadratic time effect, which indicated that the response rates significantly decreased over the first 12 months of the trial (p < .001, Figure 2).

**Table 2:** Statistically significant predictors of web-based response from generalized linear mixed model*

Variable	Odds Ratio (95% CI)	P-value
Age	1.05 (1.02; 1.07)	<.001

Race/Ethnicity		
African American vs. Caucasian	.76 (.57; .99)	.046

Education		
High school or lower vs. Graduated from college or higher	.54 (.40; .71)	<.001
Some college vs. Graduated from college or higher	.70 (.54; .91)	.008

Military status		
Active vs. Reserve	.68 (.56; .81)	<.001

Smoke Prior to Army		
No vs. Yes	1.69 (1.41; 2.03)	<.001

BMI		
Increasing 1 unit	.97 (.94; 1.00)	.027

Physical examination		
No vs. Yes	.70 (.54; .90)	.006

*The fitted model included all potential predictors and other pre-specified explanatory variables (see text for details); results for the statistically significant predictors are reported here.

POLM, Prevention of Low Back Pain in the Military; BDI, Beck Depression Inventory; BBQ, Back Beliefs Questionnaire; STAI, State-Trait Anxiety Index; PCS, Physical Component Summary of the SF12, MCS, Mental Component Summary of the SF12, Medical Outcomes Survey 12-Item Short-Form Health Survey; BMI, Body Mass Index; PSEP, Psychosocial Educational Program.

## Discussion

The results of this analysis demonstrated that response rate to the web-based survey was significantly associated with demographic characteristics, health status, and individualized attention via additional testing. Our response rate was low compared to standard randomized clinical trials that incorporate face-to-face contact to secure follow-up data (which typically range from 80-95%[2,3]) and compared to at least one similarly designed study that depended heavily on web-based surveillance systems without direct face-to-face contact with the subject during the follow-up phase of the study [14]. The overall lower response rates observed with web-based surveillance systems compared to more traditional follow-up strategies (ie, phone, face-to-face, etc.) is likely attributable to less subject accountability during the follow-up phase of the study. Soldiers did not have face-to-face contact and accountability for survey completion following the initial training phase of the study, placing more responsibility on the individual Soldiers to complete the online surveys. Although difficult to confirm, it is likely that the geographic dispersion of Soldiers around the world, deployments to austere parts of the world with limited internet access (ie, Iraq/Afghanistan), and subsequent discharge from the Army may have also contributed to the overall decreased response rate. When using web-based surveillance systems, follow-up rates may be further enhanced by supplementing with traditional methods such as phone call centers and querying available databases for health care utilization related to LBP. These combinations of multiple follow-up strategies have the potential to increase overall response rates compared to any single strategy alone but need further testing before firm recommendations can be made.

Among health status factors, a Soldier's smoking status was a significant predictor of response rates to the web-based surveillance system. Those who did not smoke prior to entering the Army had a 1.7 times odds of response compared to those who smoked prior to entering the Army (p <.001). This means Soldiers who smoked had 42.0% lower odds of response compared to those who did not smoke. It is possible that smoking status may be related to other measures of health, yet smoking still emerged as an independent predictor of response rates despite controlling for these factors. Perhaps Soldiers who smoke are less inclined to appreciate the importance of health-related research. This finding is particularly relevant for the POLM trial because 35.8% of Soldiers in this study classified themselves as smokers, defined as individuals who had smoked at least 100 cigarettes in their lifetime. The POLM trial data are consistent with a recent study demonstrating that 32.2% of military personnel are smokers [15]. In contrast, approximately 21% of the general public among a similar age group are smokers [15]. This indicates that smoking status may need to be considered during study planning, particularly for powering large clinical trials in which the primary outcome may be an infrequent occurrence or when performing studies with a high preponderance of smokers.

Another significant predictor of response rates in the POLM trial was military status, defined as whether the Soldier was in an "active duty" or "reservist" status. Active duty Soldiers had 15.6% response rate compared to 23.5% of those in the reserves (<.001). Although this consideration may have limited applicability beyond the military population, this distinction appears to be an important consideration for designing trials that include military subjects. The reason for the discrepancy in response rates between active duty and reservists is unclear; however, there are several possible explanations for this finding. Many of the training requirements for reservists are completed individually online via a variety of distance-based training platforms given their part time status and geographic separation from their active duty Army counterparts. As a result, the increase in their response rates could be partially explained by their increased familiarity with online training. Although purely speculative, perhaps reservist personnel also tend to be more self-motivated to complete training requirements because they are more accustomed to not having significant day-to-day oversight and accountability for completing their training requirements, which is closely aligned with the methodology used to administer the web-based follow up for the POLM study. Alternatively, active duty Soldiers tend to complete training requirements in groups settings within environments that have more direct monitoring and accountability. These differences may influence this group to be less likely to respond to the follow-up surveys in the absence of direct accountability.

In addition, higher educational levels were associated with increased response rates on the follow-up survey. Specifically, 20.8% of Soldiers with at least some college education responded compared to 14.6% of those who only completed high school (p <.001). College graduates had the highest response rates at 30.9%. Individuals with a high school education or lower were only .5 times as likely to respond as those with some college, whereas those with some college education were .7 times as likely to respond as college graduates. These results are not surprising since one might suspect that individuals with higher levels of education may have more intrinsic motivation and are therefore more likely to respond [16]. It is also possible that these individuals have a better appreciation for the value of health-related research and importance of subject participation. Furthermore, the increased response rates among Soldier who had completed at least some college may be related to increased computer literacy, which could certainly influence response rates given the web-based platform utilized to assess follow-up in the POLM trial.

Previous research has demonstrated that increased subject attention may enhance follow-up rates in clinical trials, regardless of the follow-up mechanism that is used [4]. To examine the potential for increased attention to enhance response rates in the POLM trial, we examined group and individualized attention. Subjects in the PSEP group who received the additional back education class in a group setting were classified as having received additional group attention, whereas Soldiers randomized to receive the physical/USI examination were classified as having received additional individual attention. The results of this study reinforce conclusions from the existing literature that increased attention during trials may enhance response rates, even when the extra attention is not directly related to completing follow-up procedures. However, a statistically significant enhancement in response rates was only observed among Soldiers who received increased individualized attention. For example, Soldiers receiving individualized attention had response rates of 24.0% compared to 18.4% among those who did not (p = .009). In contrast, receiving group attention was not associated with significant improved response rates. Soldiers receiving PSEP had response rates of 19.1% compared to 18.7% among those who did not (Table 1). Soldiers receiving both PSEP and USI had response rates of 26.4% compared to 21.4% among those receiving USI only, but this difference was not statistically significant due to small sample sizes.

Our results are in contrast to the findings from previous studies that have found increased attention in group settings to be associated with increased follow-up rates [5]. One possible explanation for this discrepant finding is that a large majority of the training completed by Soldiers in the military is done in group settings. Thus it is possible that Soldiers in the PSEP group may have perceived the back education class as an additional training burden, as opposed to value added training designed to improve their ability to cope with back pain. Additionally, the back education class was completed on a Saturday morning outside of the normal training syllabus, which could have been perceived in a more negative light. On the other hand, the physical examinations were substituted for another training requirement rather than additive, increasing the likelihood that Soldiers perceived receiving the examination as a "good deal" because they were exempt from that morning's physical training. Additionally, individualized attention from the examination may have peaked the Soldiers interest and personal appreciation for the study, further building rapport between the Soldiers and study staff, increasing their "buy-in" to the study. Designing trials that include individualized attention is an important consideration for improving response rates in trials, which helps to improve precision of the results and increase overall generalizability of the findings. However, more attention must be paid to the type of attention that provides maximal improvement in response rate, instead of the assumption that any additional attention is value added.

Several limitations for this analysis should be considered. Despite achieving statistical significance, it's possible that some of the findings may be spurious, as evidenced by the questionable meaningfulness of the effect sizes among some of the significant findings, predominantly age, race/ethnicity, and BMI. The confidence intervals of the odds ratios according to our data approximated a value of 1.0, which is equivalent to no increase or decrease in odds of response, thus negating the potential meaningfulness of these findings. As an example, age emerged as a significant predictor of response rates, yet the mean age among responders was 22.8 compared to 21.8 years of age among non responders, resulting in an odds ratio of 1.1. Although this result was statistically significant, one year in age difference does not appear to be a material finding that might inform the design of future trials. Similar findings were observed for both race/ethnicity and BMI. These small but statistically significant effects can likely be attributed to fact that the original POLM study was powered on the primary aim of detecting future episodes of back pain in the 2 years following completion of training. This may have resulted in an increased chance for Type I error in this secondary analysis.

This study reported predictors of response to a web-based survey using a dichotomous outcome to represent response rate. This decision was made because the primary outcome of the trial is a dichotomous measure (occurrence of low back pain) and we wanted these analyses to be parallel. Our additional analysis showed that response rates significantly decreased over time, which was an expected outcome that is typical in clinical trials. Also, it would be interesting to assess whether internet access was a barrier for some of the Soldiers in this study, in particular those who were deployed in remote settings around the world. However, this information was not available to us, hence we can only speculate that response rates may be lower for those Soldiers who did not internet access during the follow-up phase of the study. Future studies might also examine whether other contemporary methods of communication (ie, SMS text messaging, social media, etc.) might be more effective than email in securing follow-up [17].

Another limitation is that the subjects in this trial were more homogenous compared to the general population. Many of the Soldiers' eating habits, activity levels, and work environments are nearly identical because of the more controlled environment within the military. Similarly, subjects in the military have been shown to have similar psychological profiles [18]. As a result, these factors would not have had the opportunity to compete for explaining additional variance in the response rates, even if some relationship might exist in a more heterogeneous general public. This is potentially the reason why the psychological factors did not remain in the final regression model as predictors of response rates. Finally, the participants in this study were training to become combat medics. One might expect that their response rate would be higher than Soldiers in non-medical fields, similar to how medical personnel demonstrate higher response rates compared to subjects in the general population [19]. However we had no comparison group in the current study so we can only speculate that these follow up rates might be higher than if this study targeted subjects in the general population.

## Conclusions

Understanding which factors are associated with response rates can help to improve follow-up by informing the design of clinical trials and improving our understanding of the effectiveness of web-based surveillance systems in large clinical trials among a highly geographically dispersed subject pool. Additional attention during a trial may improve response rates, but optimal strategies have yet to be identified. Future studies should consider how to best incorporate individualized attention within clinical trials to increase response rates. Researchers should also monitor other predictors of follow-up rates identified in this analysis within their clinical trials so that any deferential influence of these factors in response rates can be considered when interpreting the results of their studies.

## List of abbreviations used

POLM: Prevention of Low Back Pain in the Military Trial; BDI: Beck Depression Inventory; BBQ: Back Beliefs Questionnaire; STAI: State-Trait Anxiety Index; SF12: Medical Outcomes Survey 12-Item Short-Form Health Survey; BMI: Body Mass Index; CSEP: Core Stabilization Exercise Program; TEP: Traditional Exercise Program; AIT: Advanced Individualized Training; PSEP: Psychosocial Educational Program; USI: Ultrasound Imaging; LBP: low back pain; RCT: randomized controlled trial; FPQ-III: Fear of Pain Questionnaire, FABQ: Fear-avoidance Beliefs Questionnaire; PCS: Physical Component Summary scale; MCS: Mental Component Summary scale; ODI: Oswestry Disability Inventory; NPRS: Numerical Pain Rating Scale; SD: standard deviation

## Competing interests

The authors declare that they have no competing interests.

## Authors' contributions

JDC contributed by developing study design, interpreting data, and composing the manuscript. DST participated in study design and data interpretation. JJV, BFD, BJL, and GLH aided in data interpretation and manuscript composition. SSW performed the statistical analysis and helped in data interpretation. SZG aided in study design and data interpretation. All authors read and approved the final manuscript.

## Pre-publication history

The pre-publication history for this paper can be accessed here:

http://www.biomedcentral.com/1471-2474/12/132/prepub
